# Convergent evolution of reduced energy demands in extremophile fish

**DOI:** 10.1371/journal.pone.0186935

**Published:** 2017-10-27

**Authors:** Courtney N. Passow, Lenin Arias-Rodriguez, Michael Tobler

**Affiliations:** 1 Division of Biology, Kansas State University, Manhattan, Kansas, United States of America; 2 División Académica de Ciencias Biológicas, Universidad Juárez Autónoma de Tabasco, Villahermosa, Tabasco, México; Fred Hutchinson Cancer Research Center, UNITED STATES

## Abstract

Convergent evolution in organismal function can arise from nonconvergent changes in traits that contribute to that function. Theory predicts that low resource availability and high maintenance costs in extreme environments select for reductions in organismal energy demands, which could be attained through modifications of body size or metabolic rate. We tested for convergence in energy demands and underlying traits by investigating livebearing fish (genus *Poecilia*) that have repeatedly colonized toxic, hydrogen sulphide-rich springs. We quantified variation in body size and routine metabolism across replicated sulphidic and non-sulphidic populations in nature, modelled total organismal energy demands, and conducted a common-garden experiment to test whether population differences had a genetic basis. Sulphidic populations generally exhibited smaller body sizes and lower routine metabolic rates compared to non-sulphidic populations, which together caused significant reductions in total organismal energy demands in extremophile populations. Although both mechanisms contributed to variation in organismal energy demands, variance partitioning indicated reductions of body size overall had a greater effect than reductions of routine metabolism. Finally, population differences in routine metabolism documented in natural populations were maintained in common-garden reared individuals, indicating evolved differences. In combination with other studies, these results suggest that reductions in energy demands may represent a common theme in adaptation to physiochemical stressors. Selection for reduced energy demand may particularly affect body size, which has implications for life history evolution in extreme environments.

## Introduction

Convergent evolution, where disparate lineages exposed to similar environmental conditions independently evolve similar phenotypes, is a central theme in evolutionary diversification [1–3]. Convergence has been documented in a wide variety of traits and in response to different sources of selection [4–6]. Although convergent evolution is frequently interpreted as evidence for the deterministic nature of natural selection, adaptation to similar environmental conditions does not consistently lead to identical evolutionary outcomes, with individual lineages sometimes diverging in unique, nonconvergent ways [7–9]. One reason for nonconvergent trait evolution is that natural selection optimizes overall organismal function rather than specific traits that contribute to function [8, 9]. Hence, there may be alternative phenotypic modifications that result in similar fitness [10], and convergence at one level of organization can arise from nonconvergent changes at lower hierarchical levels [11].

The evolution of organismal energy demands is an excellent example of how similar functional changes can arise through different mechanisms. Reductions in energy demands can generally be achieved through two mutually non-exclusive mechanisms: Organisms can evolve a smaller body mass, which decreases costs associated with growth and maintenance [12]. Alternatively, they can evolve lower metabolic rates independent of body size, thus reducing their overall metabolic expenditure [13]. We were interested in testing whether colonization of and adaptation to extreme environments leads to convergent shifts in organismal energy demands, and whether the underlying mechanisms (i.e., relative effects of body mass *vs*. metabolic rate) are contributing equally to variation in energetic demands among populations. Life in extreme environments is often associated with energetic costs. Some extreme environments, such as the deep sea and caves that are subject to periodic flooding, exhibit high spatial or temporal variability in resource availability, and organisms in such environments have been documented to evolve large body size to increase metabolic efficiency and starvation resistance, allowing them to cope with periods of energy limitation [14–16]. In other extreme environments, organisms face perpetually low availability or quality of food, engage in coping strategies that affect rates of resource acquisition, or experience increased maintenance costs in the presence of environmental stressors [17]. Life history theory predicts that all of these factors constrain the amount of energy available for reproduction and exert selection for a reduction of organismal energy demands, ultimately allowing for the maximization of relative energy allocation to the production of offspring [18]. There is also some empirical evidence supporting the hypothesis that some extreme environmental conditions favour the reduction of energy demands [19, 20].

Our study focused on the *Poecilia mexicana* species complex (Poeciliidae), in which multiple lineages have independently colonized toxic, hydrogen sulphide (H_2_S) rich springs across four river drainages in southern Mexico [21, 22]. Sulphide springs in this region exhibit average H_2_S concentrations between 20 and 200 μM (with peak concentrations reaching over 1000 μM) [21], which are all well above the toxicity threshold for most metazoans [23]. In addition, sulphide springs are characterized by hypoxia, reduced pH, and increased levels of conductivity as compared to adjacent non-sulphidic habitats [21]. Sulphide spring populations of *Poecilia* exhibit adaptive modifications of behavioural, physiological, morphological, and life-history traits, which have largely evolved in convergence [24]. In addition, sulphide adapted populations are reproductively isolated from adjacent, ancestral populations residing in non-sulphidic environments [25], despite close spatial proximity and a lack of major physical barriers preventing fish movement [25, 26]. Based on theoretical and empirical considerations, the presence of H_2_S should affect organismal energy budgets in multiple ways [27]. H_2_S causes and aggravates hypoxia in natural environments [28], forcing fish to trade-off benthic foraging with aquatic surface respiration, which mediates short-term survival [29]. In addition, H_2_S constraints aerobic energy production in mitochondria [30], and fish have to rely on less efficient anaerobic metabolism for the generation of ATP [31]. Finally, tolerating exposure to H_2_S requires active detoxification [31], and enzymatic sulphide oxidation to a less toxic form requires energy [32].

**Fig 1:**
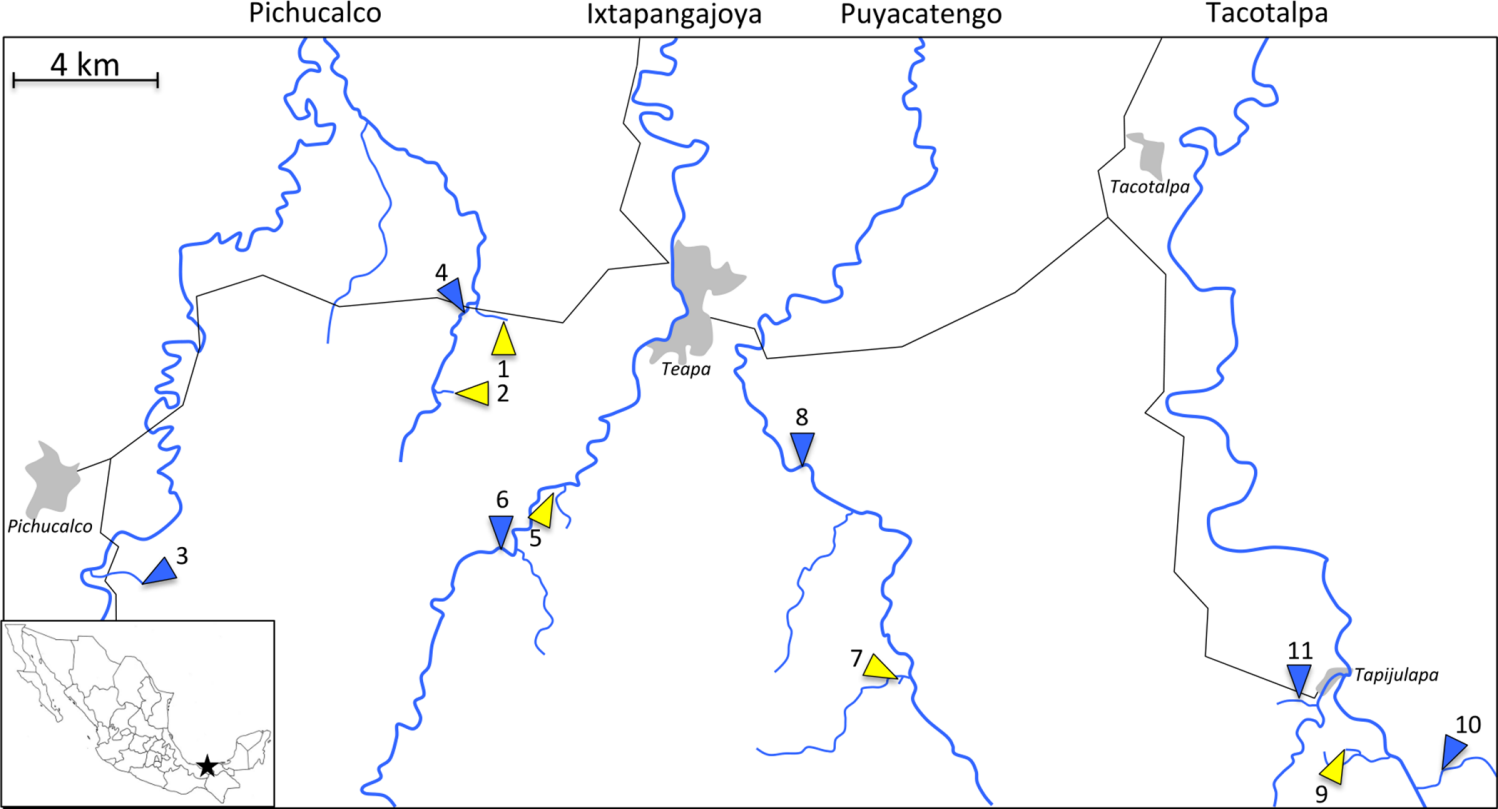
Map of the study region. Depicted are the localities of focal sulphidic (yellow arrows) and non-sulphidic study sites (blue arrows) across four river drainages in southern Mexico. Numbers correspond to sites as described in Table 1. Note that the locations of major towns (shaded areas) and roads (black lines) have been added for orientation. The insert indicates the location of the study area within Mexico.

Constraints in energy acquisition and production, as well as increased maintenance costs should precipitate in selection for a reduction in energy demands in sulphidic spring populations. Indeed, we have recently shown that *Poecilia mexicana* in sulphidic and non-sulphidic caves exhibit lower energetic demands compared to ancestral populations in non-sulphidic surface habitats [33]. However, that study was conducted on a small number of focal populations collected in one drainage (Tacotalpa; Fig 1), and all extremophile populations (including the sulphidic and non-sulphidic cave) were derived from a common ancestor [34]. Consequently, it remains to be tested whether convergent evolution shapes energy demands across evolutionarily independent lineages of extremophile fish, and if it does, what relative contributions reductions in body size and mass-specific metabolic rates make to shaping among population variation in energetic demands. In this study, we investigated multiple, evolutionarily independent sulphidic and non-sulphidic populations to address the following objectives: (1) We quantified size distributions of fish populations in sulphidic and non-sulphidic habitats across four river drainages to test whether adaptation to extreme environmental conditions is associated with a reduction of body size. (2) We quantified routine metabolic rates (RMR) of wild-caught fish to test whether extremophile population exhibit consistent reductions in mass-specific energy expenditure. (3) We modelled total organismal energy demands–based on empirical data collected on body size and metabolic rate allometry–to quantify the combined effects of body mass and metabolic rate reductions in different populations. (4) We quantified RMR in common-garden-reared individuals from a subset of populations to test whether differences between sulphidic and non-sulphidic populations have a heritable basis. Our results indicated that sulphidic populations consistently exhibited reduced body sizes and in some populations also lower RMRs compared to non-sulphidic populations, resulting in convergent reductions of overall energy demands that were driven disproportionally by variation in body size. Common-garden experiments indicated that population differences in routine metabolism observed in the field were maintained in the laboratory indicating evolved differences.

## Materials and methods

### Ethics statement

Field collection permits were issued by the Mexican government (DGOPA.09004.041111.3088). All procedures employed in this study were non-lethal. This research was conducted in accordance with the recommendations in the Guide for the Care and Use of Laboratory Animals [35] and the Association for the Assessment and Accreditation of Laboratory Animal Care (AAALAC). All procedures were approved by the Institutional Animal Care and Use Committee at Kansas State University (Protocol #3418).

### Quantifying size distributions

We analysed body size distributions and routine metabolic rates (RMR) in wild-caught *Poecilia* populations from five sulphidic springs and six adjacent, non-sulphidic habitats in different tributaries of the Rio Grijalva (Pichucalco, Ixtapangajoya, Puyacatengo, and Tacotalpa river drainages; Fig 1, Table 1). To quantify body size distributions, fish were collected using seines (4 m long, 4 mm mesh width), and blotted wet weight was measured for each adult individual to the closest 0.001g. Mass-based size distributions were analysed using general linear models (GLM) with body mass (log_10_-transformed) as the dependent variable. Sex, presence or absence of H_2_S, and drainage were included as fixed factors, and population (nested in the drainage by sulphide interaction) was included as a random factor. All statistical analyses were conducted with SPSS 17 (SPSS Inc., Chicago, IL, USA) unless otherwise stated.

**Table 1:** List of populations investigated for this study. The table provides latitude and longitude of collection localities, and descriptive statistics of body masses from fish used to characterize size distributions in natural populations as well as from fish used to quantify routine metabolic rates (RMR) in wild-caught and laboratory-reared specimens. We report body masses [g] in means (± standard deviation) and ranges (in parentheses), as well as sample sizes separately for males and females of each population. Note that ID numbers correspond to the numbers in Fig 1.

ID	Site	Lat/Long	H_2_S	Size distribution		RMR wild-caught		RMR laboratory-reared	
				Females	Males	Females	Males	Females	Males
*Rio Pichucalco drainage*									
1	Baños del Azufre	17.552, -92.999	+	0.49±0.35 (0.04–1.84) N = 141	0.58±0.29 (0.04–1.73) N = 43	0.69±0.28 (0.34–1.31) N = 34	0.47±0.26 (0.20–0.87) N = 9	0.52±0.15 (0.29–0.78) N = 9	0.41±0.13 (0.22–0.60) N = 7
2	La Gloria	17.532, -93.015	+	0.59±0.99 (0.12–4.54) N = 114	0.45±0.27 (0.16–1.25) N = 43	0.96±0.61 (0.16–2.46) N = 12	0.85±0.52 (0.49–1.22) N = 2	-	-
3	Arroyo Rosita	17.485, -93.104	-	2.35±1.65 (0.21–9.04) N = 119	2.03±1.36 (0.51–6.10) N = 27	2.13±1.13 (0.30–5.52) N = 26	2.44±1.41 (1.23–6.00) N = 9	0.74±0.25 (0.48–1.13) N = 11	0.70±0.25 (0.34–1.09 N = 8
4	Rio El Azufre, west branch	17.556, -93.008	-	1.56±0.91 (0.30–4.80) N = 113	2.11±1.39 (0.59–6.06) N = 18	1.67±0.66 (0.84–3.37) N = 11	1.36±0.85 (0.46–2.79) N = 6	-	-
*Rio Ixtapangajoya drainage*									
5	La Esperanza, large spring	17.511, -92.983	+	0.35±0.20 (0.06–0.89) N = 127	0.18±0.07 (0.10–0.32) N = 19	0.79±0.25 (0.40–1.22) N = 17	0.19±0.05 (0.14–0.23) N = 3	-	-
6	Rio Ixtapangajoya	17.495, -92.998	-	1.48±1.50 (0.07–7.30) N = 152	0.60±0.48 (0.13–1.94) N = 38	1.48±0.57 (0.40–2.62) N = 26	1.06±0.77 (0.24–2.99) N = 13	-	-
*Rio Puyacatengo drainage*									
7	La Lluvia, small spring	17.464, -92.895	+	1.42±1.42 (0.03–6.63) N = 122	0.41±0.64 (0.03–3.53) N = 107	0.83±0.71 (0.21–2.89) N = 28	0.34±0.15 (0.13–0.74) N = 16	-	-
8	Rio Puyacatengo at Vicente Guerrero	17.510, -92.914	-	1.35±1.85 (0.14–10.32) N = 167	1.44±0.83 (0.08–6.96) N = 62	1.79±0.83 (0.75–3.42) N = 14	1.44±0.83 (0.55–2.72) N = 5	-	-
*Rio Tacotalpa drainage*									
9	El Azufre I	17.442, -92.775	+	0.85±0.56 (0.16–3.00) N = 306	0.63±0.27 (0.07–1.56) N = 112	0.61±0.43 (0.12–1.47) N = 36	0.86±0.54 (0.34–2.04) N = 10	0.61±0.27 (0.30–1.17) N = 17	0.37±0.25 (0.19–0.95) N = 8
10	Arroyo Bonita	17.427, -92.752	-	1.70±1.17 (0.17–7.80) N = 247	1.54±1.22 (0.25–4.69) N = 75	1.36±0.82 (0.23–3.73) N = 30	1.06±0.50 (0.25–2.32) N = 13	0.47±0.16 (0.20–0.78) N = 9	0.73±0.69 (0.27–2.54) N = 10
11	Arroyo Tacubaya	17.454, -92.785	-	1.23±0.72 (0.15–5.23) N = 265	0.87±0.54 (0.20–2.23) N = 55	0.37±0.21 (0.14–0.79) N = 9	0.94±0.30 (0.60–1.18) N = 3	-	-

### Quantifying variation in routine metabolic rates

To quantify RMR, which is defined as the oxygen consumption of unconstrained, post-absorptive organisms capable of spontaneous movement [36], specimens were collected from focal populations and transported to a nearby field station. Fish were acclimated to standardized laboratory conditions for at least 48 hours. We used a closed chamber respirometry system to measure oxygen consumption. This approach has been widely used to quantify metabolic costs associated with a variety of traits and environmental conditions [37–39]. Methods followed protocols implemented in a previous study [33]: (1) Fish were not fed 24 hours prior to trials to assure measurements were conducted on post-absorptive individuals [40]. (2) Individuals were haphazardly chosen and placed into opaque 500mL respirometry bottles. Bottles were then placed in a water bath to minimize temperature fluctuations (average ± SD: 25.1 ± 1.9°C). For acclimation to experimental conditions, fish were left undisturbed in the bottles with continuous aeration for at least 12 hours. (3) After acclimation, bottles were flushed with fresh aerated water to remove metabolic waste products that could affect metabolism [40] and capped with a lid that had a hole drilled in the top to allow for the insertion of a YSI ProODO optical dissolved oxygen probe (YSI Inc., Yellow Springs, OH, USA). Plumbers putty was fitted around the probe to prevent gas exchange during the trial. Probes were set to measure the dissolved oxygen concentration at 10-second intervals. Note that all trials were conducted in absence of H_2_S even for sulphidic populations, because the reactivity of H_2_S with oxygen in aqueous solution affects the measurement of oxygen consumption rates [41], and even fish from sulphidic sites face elevated mortality rates in presence of H_2_S without access to the water surface [unpublished data; also see [42]]. (4) After the termination of a trial, individuals were weighed and sexed (see Table 1 for descriptive statistics). For each trial, we removed outliers (random readings of zero oxygen) that were caused by instrumental error. We also removed data points from the first 60 min of each trial, as the flushing of the bottle with fresh water and the installation of the probe may have caused erratic fish activity [40]. Because fish metabolic rates may be affected by reduced ambient oxygen concentrations [37], we only included data points measured at dissolved oxygen saturations ≥70%. Routine metabolic rate (in mgO_2_/hour) was then calculated for each individual as the slope of a regression (multiplied by the volume of water in the bottle) with oxygen concentration as the dependent variable and time as the independent variable (mean *R*^2^ = 0.99). Routine metabolic rate data (log_10_-transformed) were analysed using GLM with sex, presence or absence of H_2_S, and drainage as fixed factors, and population (nested in the drainage by sulphide interaction) as a random factor. Temperature and mass (log_10_-transformed) were included as covariates in all models. Three-way interaction terms were not significant (*F* ≤ 1.62, *P* ≥ 0.19) and were excluded from the final model.

### Simulating total metabolic rates

To test how variation in body size and RMR interact to shape organismal energy demands, we modelled total routine metabolic rates for individuals in each population based on the empirical data on size distributions and allometric metabolic rate functions [33]. For each population, we first resampled size distributions based on the field data 1000 times. For each resampled individual, total metabolic rate was calculated as log_10_(*MR*_tot_) = b*log_10_(mass)+*a*, where *b* was the slope and *a* the intercept of a regression describing the relationship between mass and metabolic rate for each population. To account for uncertainty associated with the estimation of slopes and intercepts, values for *b* and *a* were randomly chosen from within the 95% confidence interval of each parameter. The simulated values of total routine metabolic rate consequently represent estimates of the energy demand of average individuals in each population, taking into account within and among-population variation in both body mass and metabolic rate allometry. Simulated total metabolic rates were analysed using GLM with presence or absence of H_2_S in natural populations and drainage as fixed factors, and population (nested in the drainage by sulphide interaction) as a random factor.

**Fig 2:**
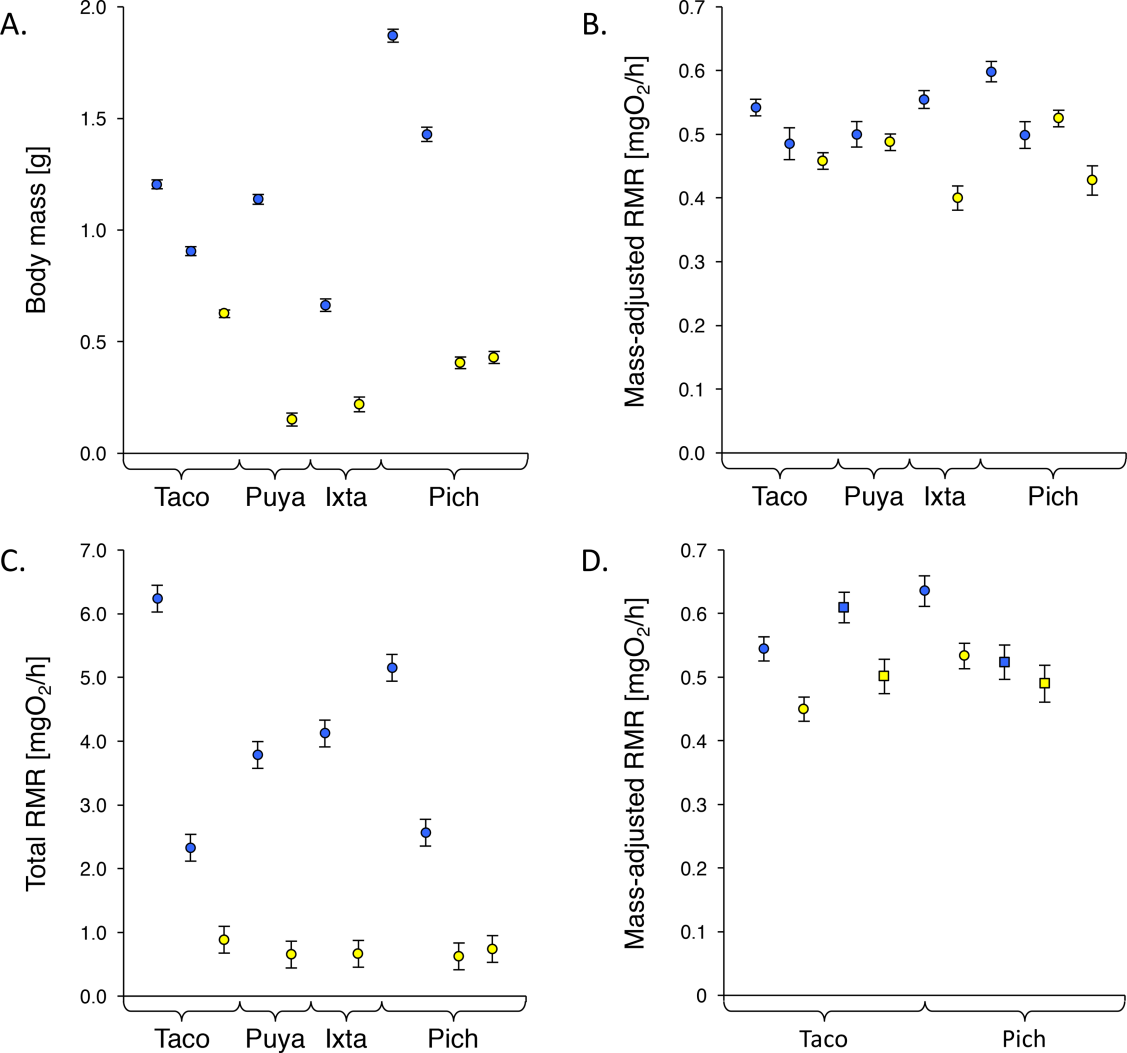
Population differences in body size, mass-adjusted routine metabolic rates, and total organismal energy demands. Variation in (a) body size, (b) mass-adjusted routine metabolic rate, and (c) total routine metabolic rate. Depicted are estimated marginal means (EMM ± standard error) based on analytical models presented in Table 2. Populations are organized by river drainage; blue symbols represent non-sulphidic populations, yellow symbols sulphidic ones. Mean values of covariates used for the calculation of EMM of mass-adjusted routine metabolic rate were as follows: mass = 0.83 g; temperature = 25.1°C. (d) Differences in mass-adjusted routine metabolic rate between wild-caught (circles) and laboratory-reared (squares) fish from a subset of populations. Mean values of covariates used for the calculation of EMM were as follows: mass = 0.72 g; temperature = 24.2°C.

In addition, we tested whether among population variation in total organismal energy demands was primarily driven by variation in body size or mass-adjusted RMR. To do so, we first calculated the average of total organismal energy demands, body mass, and mass-adjusted RMR for each population (as depicted in Fig 2A–2C). We then partitioned the variation in total organismal energy demands (as the dependent variable) with respect to body mass and mass-adjusted RMR (independent variables) using the *varpart* command implemented in the R package vegan [43]. This analysis partitions variation in the dependent variable into the fractions uniquely explained by each of the independent variables as well as their joint fraction, which represents the fraction of variation of the dependent variable that may indifferently be attributed to either of the independent variables [44].

### Common-garden experiment

Metabolic rate variation in wild-caught fish may merely reflect plastic responses to exposure to different environments. Hence, we conducted a common-garden experiment to test whether variation in mass-adjusted RMR between sulphidic and non-sulphidic populations has a heritable basis. Fish were collected from a subset of sites (Table 1) and transported to the laboratory at Kansas State University. Juveniles born to wild-caught mothers were isolated in family groups and raised to adulthood (standard length >30 mm). All fish were kept under non-sulphidic conditions with a 12:12 hour light:dark cycle and a constant temperature of 25°C. The experimental protocol for metabolic rate measurements was identical to the one for wild-caught fish (average temperature during trials ± SD: 24.2 ± 1.1°C). Data on metabolic rates (log_10_-transformed) from common-garden reared fish were combined with data from wild-caught fish from the matching sites and used as the dependent variable in a GLM. We included sex, presence or absence of H_2_S, drainage, and rearing environment (i.e. wild-caught *vs*. laboratory-reared) as independent variables. Temperature and mass (log_10_-transformed) were included as covariates. Three-way and four-way interactions were not significant (*F* ≤ 3.26, *P* ≥ 0.08) and thus excluded from the final model.

## Results

We measured body mass for *N* = 2,472 individuals collected across all sites (see Table 1 for descriptive statistics). All terms included in the GLM were highly significant, but the presence or absence of H_2_S explained the largest portion of variation in mass (Table 2A). As predicted, individuals from sulphidic populations were consistently smaller than those from non-sulphidic populations of the same drainage (Fig 2A). We found that the magnitude of difference between sulphidic and non-sulphidic populations varied among the different drainages, and that there was also significant variation among the specific populations analysed. Males were generally smaller than females, which is likely a consequence of male–but not female–poeciliids exhibiting determinate growth [42, 45]. Note that sample sizes were typically lower for males than females, which is reflective of the highly female biased sex ratio in natural populations [42].

**Table 2:** Results of general linear models analysing variation in body size and metabolic rates. (a) Comparison of body mass among populations. (b) Comparison of routine metabolic rates in wild-caught individuals. (c) Comparison of simulated total metabolic rates. (d) Comparison of routine metabolic rates in wild-caught and common-garden raised individuals for a subset of populations. Note that the effect size for each of the terms in a model was estimated by use of partial eta squared (*η*_p_^2^). Relative variance was calculated as the partial eta squared for a particular term divided by the maximum partial eta squared in the model.

Variable	*df*	*F*	*P*	η_p_^2^	Relative variance
a. Body mass					
Sex	1	51.192	<0.001	0.020	0.133
Drainage	3	45.234	<0.001	0.052	0.342
H_2_S	1	443.587	<0.001	0.153	1.000
Population (Drainage × H_2_S)	4	10.600	<0.001	0.017	0.111
Sex × Drainage	3	14.123	<0.001	0.017	0.111
Sex × H_2_S	1	10.467	0.001	0.004	0.028
Drainage × H_2_S	3	26.663	<0.001	0.032	0.206
Sex × Drainage × H_2_S	3	22.882	<0.001	0.027	0.178
b. Routine metabolic rate (wild-caught fish)					
Mass (log_10_-transformed)	1	637.391	<0.001	0.670	1.000
Temperature	1	185.013	<0.001	0.371	0.554
Sex	1	0.585	0.445	0.002	0.003
Drainage	3	2.800	0.040	0.026	0.039
H_2_S	1	22.439	<0.001	0.067	0.100
Population (Drainage × H_2_S)	3	7.870	<0.001	0.070	0.104
Sex × Drainage	3	2.080	0.103	0.019	0.028
Sex × H_2_S	1	0.383	0.536	0.001	0.001
Drainage × H_2_S	3	5.862	0.001	0.053	0.079
c. Simulated energy demand					
Drainage	3	5.305	0.001	0.001	0.005
H_2_S	1	3038.772	<0.001	0.217	1.000
Population (Drainage × H_2_S)	3	103.974	<0.001	0.028	0.129
Drainage × H_2_S	3	6.352	<0.001	0.002	0.009
d. Routine metabolic rate (laboratory-reared fish)					
Mass (log_10_-transformed)	1	210.571	<0.001	0.475	1.000
Temperature	1	58.632	<0.001	0.201	0.424
Sex	1	2.005	0.158	0.009	0.018
Drainage	1	0.539	0.464	0.002	0.005
H_2_S	1	13.940	<0.001	0.056	0.119
Wild/Laboratory	1	0.007	0.934	0.000	0.000
Sex × Drainage	1	3.947	0.048	0.017	0.035
Sex × H_2_S	1	1.029	0.311	0.004	0.009
Sex × Wild/Laboratory	1	0.715	0.399	0.003	0.006
Drainage × H_2_S	1	0.580	0.447	0.002	0.005
Drainage × Wild/Laboratory	1	12.766	<0.001	0.052	0.109
H_2_S × Wild/Laboratory	1	0.482	0.488	0.002	0.004

We measured RMR in *N* = 332 wild-caught individuals from 5 sulphidic and 6 non-sulphidic populations. Body mass and temperature explained most of the variation in RMR, but RMR also varied among populations and drainages (Table 2B). Most importantly, individuals from sulphidic habitats overall exhibited lower mass-adjusted RMR than those from non-sulphidic habitats. However, we found that this pattern was only pronounced in the populations of the Ixtapangajoya and Pichucalco river drainages (Fig 2B), which explains the significant drainage by habitat type interaction term in the model.

Simulations of total organismal energy demands indicated that the presence of H_2_S explained the bulk of variation in the dataset (Table 2C), and fish from sulphidic populations exhibited substantially lower energetic demands (Fig 2C). Nonetheless, the magnitude of differences between sulphidic and non-sulphidic populations varied among drainages (Fig 2C). Among population variation in total organismal energy demands was primarily associated with body size, which uniquely accounted for 26% of the observed variation. Variation in RMR alone only accounted for 4% of variation in total organismal energy demands, while the joint effect of the two predictor variables was 41%.

Comparison of RMR between wild-caught and common-garden reared individuals from a subset of populations indicated that energy consumption rates were higher in the wild than the laboratory for populations from the Tacotalpa drainage, but lower in the wild than in the laboratory for the Pichucalco populations, as indicated by the significant interaction term between drainage and rearing environment (Fig 2D, Table 2D). Nonetheless, differences in energy consumption rates between sulphidic and non-sulphidic sites were maintained in the laboratory, with sulphidic fish having lower mass-adjusted RMR than non-sulphidic fish (Fig 2D). Other than mass and temperature, habitat type of origin actually explained the most variation in the model.

## Discussion

We investigated the energy demands of organisms in extreme environments by comparing body size and metabolic rate variation among locally adapted fish populations inhabiting replicated sulphidic and non-sulphidic habitats. We found that fish from toxic sulphide springs were both smaller and had lower mass-adjusted RMRs, even though differences in RMRs were not pronounced in all river drainages. Simulating total metabolic rates based on variation in body mass and RMR allometry indicated pronounced reductions in overall energy demands of extremophile populations, revealing a pattern of convergent evolution across evolutionary independent lineages. Among population variation in simulated energy demands was disproportionally associated with variation in body size and less so by variation in RMR, perhaps suggesting evolutionary change in body size faces fewer evolutionary constraints than modification of metabolic rates. Nonetheless, the maintenance of population differences in mass-adjusted RMR of laboratory-reared fish supports the conclusion of evolved population differences in metabolism.

### Variation in body size

Fish in sulphide springs consistently exhibited smaller body size than ancestral populations in non-sulphidic habitats, which is consistent with the hypothesis that selection in H_2_S-rich environments should favour reduced energetic demands. It is important to note, however, that it is difficult to determine the ultimate causes of body size variation among populations and to discriminate between effects of energy limitation and other sources of selection. Selective regimes between adjacent sulphidic and non-sulphidic habitats are notoriously complex, with multiple abiotic and biotic factors coinciding along the environmental gradient connecting the two [27, 46]. Consequently, other sources of selection may drive or at least contribute to body size differences among populations. It is unlikely that avoidance of H_2_S toxicity contributes to the reduced body size in sulphide springs, because H_2_S toxicity should select for a smaller surface:volume ratio and hence a larger body size in sulphide spring populations [47, 48]. However, the presence of H_2_S is also correlated with severe hypoxia [28, 49], and limited oxygen availability has been shown to constrain growth in fish [50] and other organisms [51]. In addition, high population densities in sulphidic environments [52, 53] could lead to density-dependent growth as a result of intraspecific competition for resources [54].

Selection may also directly act on life history characteristics independent of energetic constraints and physiochemical stressors. For example, differences in predation regimes between habitat types could exert selection on size at maturity [5]. While the virtual absence of fish predators in sulphide springs [55] would predict larger body size in sulphidic habitats [5], it remains to be tested whether predation by birds [56] and potentially some insects [57, 58] could favour smaller body size in sulphide spring populations. Finally, body size differences among populations could also be the consequence of sexual selection. Sexual harassment in sulphidic populations with high population densities may limit resource acquisition rates [59], or reductions in choosiness under the stressful environmental conditions may relax sexual selection for large sized mates [60]. Future studies will consequently need to focus on disentangling the potential contribution of co-varying abiotic and biotic factors in body size variation among populations and habitat types in this system [12, 13]. It also remains to be tested whether population differences in body size observed in nature have a heritable basis, or whether they are entirely plastic and governed by ambient ecological conditions.

### Variation in routine metabolic rates

Variation in routine energy consumption rates among organisms is primarily explained by body mass and temperature [61]. Nonetheless, both plastic and genetic factors can cause deviations from mass- and temperature-dependent metabolic scaling relationships [13], and analysis of common-garden reared individuals uncovered evidence for both. Effects of plasticity were evident, as common-garden raised fish exhibited different mass-adjusted RMR compared to wild-caught individuals. At the same time, differences in mass-adjusted RMR between sulphidic and non-sulphidic populations were partially driven by genetic variation among populations, because sulphide spring fish retained lower oxygen consumption rates even when raised under standardized, non-sulphidic conditions. Consequently, reductions in routine energy consumption rates documented in the wild-caught fish may in part be driven by evolutionary differentiation among proximate populations that are exposed to contrasting environmental conditions, although it remains unclear how epigenetic effects may have influenced metabolic rate variation in our experiment [62].

Several non-mutually exclusive, proximate mechanisms could cause the documented reduced mass-adjusted RMR in sulphide spring environments. (1) Variation in RMR may simply reflect differences in activity rates or other aspects of behaviour [63, 64]. Since metabolic rates were quantified using closed chamber respirometry, individual fish were capable of spontaneous movements, and population differences in general activity patterns or the expression of costly behaviours could consequently shape variation in RMR. A previous study that measured activity levels revealed that fish from sulphidic environments tended to exhibit higher activity rates than fish from the non-sulphidic environments, and activity rates did not accurately predict RMR [33]. However, there is evidence that fish from sulphidic environments have reduced costly behaviours associated with aggregation and mating [65, 66], and they are less bold compared to their non-sulphidic counterparts [67]. These observations indicate that the reduction of routine energy consumption may indeed be a consequence of behavioural population differences. (2) Reduced mass-specific RMR may be related to a reduced investment into energetically expensive tissues [68]. The relative size of costly organs–such as the brain and digestive organs–has been shown to be correlated with whole organism metabolic rates [69, 70]. In addition, the brain, organs associated with the digestive tract, and circulatory tissues have particularly high repair and maintenance costs, which may be exacerbated in the presence of physiochemical stressors and disproportionally influence metabolism [71, 72]. Indeed, sulphide spring fish exhibit significantly smaller brain sizes [72] and shorter gastrointestinal tracts [73] as compared to non-sulphidic spring fish, which could be associated with the observed differences in energy consumption rates. (3) Reduced metabolic rates in sulphide spring fish may be a consequence of physiological modifications that have occurred in direct response to selection from the presence of H_2_S. H_2_S is potent respiratory toxicant that directly interferes with mitochondrial function and aerobic ATP production [30]. At least in some sulphide spring populations investigated here (Pichucalco and Puyacatengo drainages), there is evidence for adaptive modification of cytochrome c oxidase, which represents the primary toxicity target of H_2_S and the enzyme responsible for oxygen consumption by mitochondrial oxidative phosphorylation [74]. Modified cytochrome oxidase in sulphide spring populations of *P*. *mexicana* allows for the maintenance of aerobic ATP production in presence of H_2_S, but it remains unclear whether there are any effects on mitochondrial oxygen consumption rates in the absence of H_2_S. (4) Reduced metabolic rates may be a consequence of physiological modifications in response to variation in oxygen or energy availability among habitat types, as oxygen limitation [75] as well as quantitative [76] and qualitative [77] differences in diets can affect metabolic expenditure. Sulphide springs are extremely hypoxic [49], and genes associated with anaerobic metabolism are up-regulated in natural populations [31]. In addition, sulphidic and non-sulphidic populations differ in both resource acquisition rates [29] and dietary resource use [78]. (5) Reduced RMR in the sulphide-adapted fish may be a consequence of our experimental design that measured oxygen consumption rates in absence of H_2_S. It is possible that routine metabolic rates in presence of H_2_S are substantially higher due to costs associated with H_2_S detoxification and tissue repair. Thus, organismal energy demands *in situ* may not differ significantly between sulphidic and non-sulphidic populations, or they may even be higher in fish from H_2_S-rich environments. Nonetheless, the reduced energy demand in sulphide spring fish that we documented in absence of H_2_S may be adaptive, if it allows for the accommodation of costs associated with detoxification and maintenance without exceeding the maximal metabolic rate when H_2_S is present.

### Overall reductions in energy demands

While most inferences about energy demands of extremophiles have solely been drawn from analyses of metabolic rates, our study suggests that variation in body size and metabolic rates should be considered jointly. Variance partitioning indicated that variation in organismal energy demand among populations was disproportionally associated with body size. Modulation of body size upon colonization of extreme environments therefore is an important contributor to shaping organismal energy demands. Nonetheless, it is important to emphasize that evolutionary change in mass-adjusted metabolic rates was not negligible and complementary to body size reductions in populations from H_2_S-rich habitats. This is highlighted by a positive correlation between the population-specific deviation in body size and in mass-adjusted metabolic rates from the among population average (Pearson correlation: *r* = 0.662, *P* = 0.027). In other words, populations with lower than average body sizes also exhibited lower than average mass-adjusted metabolic rates, indicating that modification of both traits contribute to evolutionary reductions of energy demands.

Overall, our data on body size variation and oxygen consumption rates are consistent with theoretical considerations that predict reductions in energy demands of extremophiles in response to selection mediated by high maintenance costs and/or consistently low resource availability [17, 18]. They are also consistent with empirical evidence from other organisms adapted to extreme environments found in some caves, the deep sea, as well as hypersaline and sulphidic habitats [33, 40, 79, 80], perhaps suggesting that adaptive modifications of energy budgets are a common theme in adaptation to extreme environments. Interestingly, reductions of energy demands in sulphide spring fish also parallel divergence in the expression of other costly traits [24]. For example, previous studies have documented shifts in reproductive life-history traits from producing many small offspring in non-sulphidic populations to few, large offspring in sulphidic populations [23, 81], a reduced investment into energetically costly organs [82], and a reduction in costly behaviours [64, 65]. Collectively, these results bolster the notion that bioenergetics represents important nexus to understand evolution of complex phenotypic traits in extreme environments.
